# The acceptability to patients with macular disease to have retreatment decisions being made by artificial intelligence

**DOI:** 10.1038/s44440-025-00011-7

**Published:** 2026-01-19

**Authors:** Sarah Clinton, Alexander J. E. Foss, Peter S. Bloomfield

**Affiliations:** 1 Macular Society, Crown Chambers, South Street, Andover, Hampshire UK; 2 Nottingham University Hospitals NHS Trust, Hucknall Road, Nottingham, Nottinghamshire UK

**Keywords:** Education, Diagnosis

## Abstract

**Background/Objectives:**

**Introduction:**

Artificial Intelligence (AI) refers to a system that can take input data and subsequently provide a prediction-based output. In some instances, these systems can provide a recommendation for a clinical decision. Such systems are being developed to analyse retinal images and to determine if there is active retinal disease with a view to directly influencing treatment decisions. This study looks at whether AI-based decision making is acceptable to patients with macular disease.

**Methods:**

The Macular Society has a monthly newsletter which it sends out to its members and subscribers in which views were canvassed. They were offered participation in a conjoint analysis exploring human or AI decision making, the error rate, the time to follow up and whether the scans were double read by a human or AI tool. Options were presented in a random order and participants were asked to rank the suitability of different scenarios in order of preference.

**Results:**

The task was completed by 181 participants. The two most important factors were the error rate (*p* < 0.0001) and whether the results were being checked (*p* < 0.0001). Participants did not state a preference for the first and/or second reader being either human or AI and there was a non-significant trend for rapid turnaround.

**Conclusions:**

Patients with macular disease find AI to be acceptable in the assessment of retinal images. The most important factors to patients relate to the accuracy of the decision making.

## Introduction

Age-related macular degeneration (AMD) is the commonest cause of blindness among elderly people in the developed world. There are an estimated 67 million patients with AMD in Europe, expected to rise to 77 million by 2050 [1]. The neovascular, or wet, form of the disease requires monitoring and through treat-and-extend protocols with some treatments, patients can at times achieve a 24-week treatment interval [2, 3]. This with the aging population is causing an ever-increasing workload which is placing a strain on health care systems. Ophthalmology is the largest outpatient department of the NHS and treatment for AMD is a large proportion of this group of patients [4].

The development of modern artificial intelligence (AI) techniques such as deep learning, reinforcement learning, and large language models have presented new opportunities in clinical domains. Retinal imaging has been used in many projects exploring the predictive power of the technology and such analysis techniques are exploring the possible detection of perturbation of vision, alongside predicting the progression of conditions [5]. Similarly, automated analysis of retinal images is being used to predict risk of many other disorders, such as cardiac disease and neurological dysfunction [6]. In some instances, these techniques are more accurate than human raters of images [7]. This opens up the possibility of an automated decision-making process which can happen in real time and not require the involvement of a highly trained expert which are in limited supply.

However, it is not immediately clear whether delegating the decision-making process that directly impacts one’s clinical care to an AI system is acceptable to patients. Patient centred care was a key in the report by Robert Francis, commissioned in response to events at Stafford Hospital, with an emphasis on ensuring that the patients’ voices are heard in clinical decision making [8]. As more AI based tools are incorporated into clinical pathways, a better understanding of patient perspectives on acceptability of using said tools is crucial.

One way of determining patient preferences is to use a technique called conjoint analysis. The approach is based on the idea of a trade-off. The full concept method of analysis generates a number of profiles or scenarios with the attributes of interest for the service package or product represented. If one makes the assumptions that each factor acts independently, then one can, by careful choice of combinations use only a fraction of the possible scenarios and still be able to do a full analysis. It allows the relative importance of different attributes to be assessed and shows what features individuals are prepared to trade to obtain what they think is most important. Utility (which is a measure of desirability) scores are generated for each of the attributes and can be used to find the relative importance of each attribute. This method has been used previously to assess ophthalmic services [9–13].

## Methods

The survey was conducted using a popular survey website surveymonkey.com. This site was chosen due to its accessibility for those with visual impairments, including compatibility with screen readers. The survey was modelled on a previous conjoint survey conducted which had a strong response. Wording in the survey provided an introduction to AI to those who may be unfamiliar to this concept (Supplementary Information).

The survey was promoted through the Macular Society’s monthly e-newsletter with 82,000 subscribers, and the dedicated webpage for surveys and focus groups. The survey was live for a month, between May and June 2024.

We used the conjoint package version 1.41 developed for R by Bak and Bartlmomwicz [14] and available from The Comprehensive R Archive Network (CRAN).

We looked at four factors: -

- The initial reader and this had two levels; human or artificial intelligence.
- The error rate and this had three levels of 5%, 10% and 20%.
- Speed of result and this had three levels; within one day, within two days and within four days.
- Whether there was a second reader or checker and this had three levels; none, human and AI.

The scenarios presented for ranking described a range of orders of a first reading by AI, with either no second reading or, second reading by either a human or an AI system. The options were presented to ensure there was not an assumption of a second reader based on presentation of ‘first reading’. i.e. If the first reader was AI, the second was not automatically human or AI as a result. We chose the difference between the levels for error rate and speed of reporting to be a constant ratio rather than a constant amount.

This generates 54 possible scenarios (2 × 3 × 3 × 3). We used a partial fractional design that reduced the number of scenarios needing to be ordered to 13.

These scenarios were presented in a random order on Survey Monkey and participants were asked to rank them in order from most to least preferable. The results were stored on an Excel spreadsheet and then imported into R for analysis.

Participants were also asked about their age, gender, level of schooling (primary school, secondary school up to 16, A-levels, University and Postgraduate), whether they were undergoing intraocular injections and whether they were aware of AI (on a three point scale from have heard about it but do not understand it, aware and have some understanding, and very aware and understand it).

The utility scores for each factor level were calculated for each participant. The overall mean utility scores and their importance was calculated. Then for each factor level, a linear regression model was constructed with the part utility score for the factor level being the dependent variable and the participant age, gender, level of schooling, knowledge of AI and experience of intraocular injections entered as the independent variables. The aim of these analyses was to determine if patient specific utility values could be predicted. They were also given the opportunity to provide free text comments.

## Results

Here we report that 374 participants responded to the survey, 193 did not fully complete the survey and were excluded. In total, 181 fully completed including the ranking task. Of the 181 responders, 43% had wet AMD, 35% had dry AMD, and 7% had no macular disease.

Eighty-eight participants provided free-text comments. Seven of the comments related to finding the ranking task too difficult. The commonest comments were positive in favour of AI and saw benefits in consistency, speed of response, accuracy (though a number highlighted that this would need to be rigorously assessed and the need for further research) and freeing up time for professionals to spend more time caring. Perhaps the single most frequent comment was about AI assisting humans with humans making the final decision. There was a consistent minority view that humans should make decisions about humans.

**Fig. 1:**
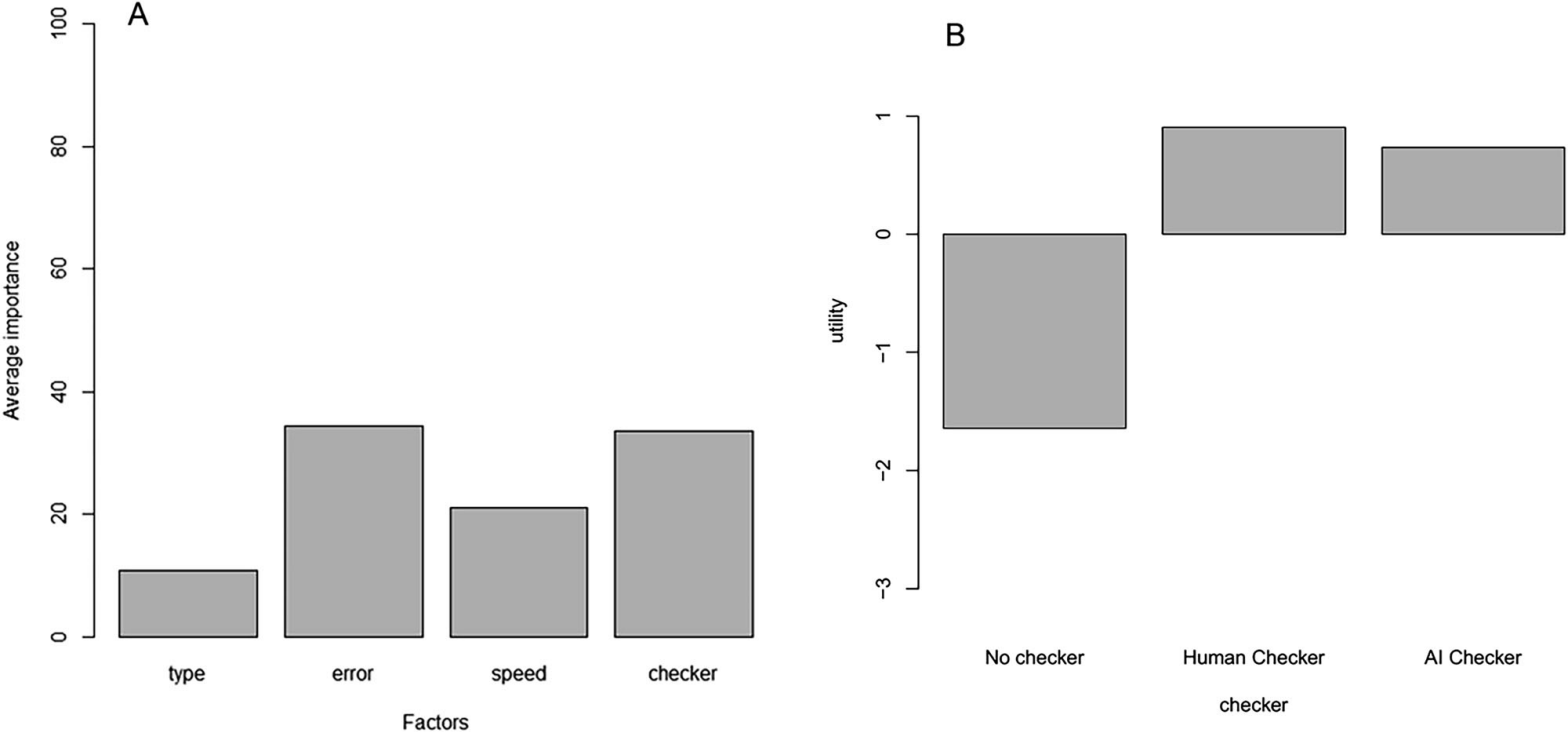
Conjoint analysis output for factors and error checking. **A** Figure showing the relative importance of the four factors. **B** The utilities values associated with having a second reader or checker showing that having the results checked would be welcomed but no significant difference between whether it was being done by either a human or by AI.

In this study, 181 individuals completed the task and the results are summarised in Table 1 and in Fig. 1A. The two most prominently valued factors were accuracy of reporting and having a second reader or checker. Overall, the least important factor was whether the initial reporting was done by a human or by AI. Similarly, when considering a second checker (see Fig. 1B), respondents did not perceive a difference in the utility value between having the second reader (checker) being human or AI.

**Table 1:** Mean utility score and importance of each factor and factor level.

Variable	Option	Mean utility score	Mean importance (%)
**First Reader**	Artificial Intelligence	–0.012	10.9
	Human Reader	0.012	
**Error rate**	5%	0.860	34.4
	10%	0.069	
	20%	–0.93	
**Time for result**	One Day	0.197	21.1
	Two Days	0.099	
	Four Days	–0.296	
**Second reader**	None	–1.65	33.6
	Human checker	0.901	
	Artificial Intelligence	0.744	
**Intercept**		7.3	

The part utility scores were saved and used as the dependent variables in linear regression models with age, gender, having treatment, knowledge of AI and level of education as the independent variables. The aim was to see whether any of these predicted participants’ values. There was a total of 11 factor levels (two for whether the initial reader was human or AI, three for spend of turnaround, three for the error levels chosen and three for checking).

No assumptions were made about the factor levels and, as can be seen for checking (Fig. 1B) there is no simple relationship between the three levels. Each factor level was entered into a linear regression as the dependent variable and age, gender, whether they had had intravitreal injections, level of education and knowledge about AI as the independent variables. Seven of the eleven has no significant associations and three did (Tables 2–4).

**Table 2:** Linear regression model predicting the utility of an error rate of 1 in 5 (top) and an error rate of 1 in 20 (bottom).

	Regression coefficient	Standard error	t-value	Probability
**1 in 5 Error rate**				
Intercept	0.73	1.65	0.44	0.66
Age	–0.02	0.14	–0.16	0.87
Gender	–0.03	0.34	–0.09	0.93
Had injections	–0.37	0.32	–1.16	0.25
Education level	0.32	0.16	2.03	0.04
Self-assessed knowledge of AI	0.07	0.30	0.25	0.81
**1 in 20 Error rate**				
Intercept	–01.68	1.70	0.99	0.32
Age	0.14	0.14	0.99	0.32
Gender	–0.15	0.35	–0.43	0.67
Had injections	0.25	0.33	0.77	0.44
Education level	–0.48	0.16	–2.98	0.003
Self-assessed knowledge of AI	0.26	0.31	0.84	0.40

**Table 3:** Linear regression model predicting the utility of an error rate of AI checker.

	Regression coefficient	Standard error	*t-*value	Probability
**Intercept**	0.32	1.20	0.27	0.79
**Age**	–0.04	0.10	–0.48	0.63
**Gender**	0.61	0.25	2.47	0.01
**Had injections**	0.22	0.23	0.96	0.34
**Education level**	–0.13	0.11	–1.18	0.24
**Self-assessed knowledge of AI**	–0.53	0.22	–2.40	0.017

**Table 4:** Linear regression model predicting the utility for no checker.

	Regression coefficient	Standard Error	*t* value	Probability
**Intercept**	0.41	1.49	0.27	0.78
**Age**	–0.06	0.12	00.48	0.63
**Gender**	–0.45	0.31	–1.48	0.14
**Had injections**	–0.12	0.29	–0.42	0.68
**Education level**	0.23	0.14	1.66	0.10
**Self-assessed knowledge of AI**	0.65	0.27	2.37	0.019

AI would appear to be very acceptable but with a non-significant trend in favour of a human making the decisions (mean utility score for AI, and human respectively: –0.012, 0.012). Those who claimed to know most about AI trusted it least and women were less trusting than men. Table 2 showed a negative regression coefficient for using AI as a checker and awareness and a corresponding positive coefficient between knowledge and no checker at all (Table 4).

## Discussion

The result of this conjoint analysis shows that participants were most concerned about the accuracy of test interpretation as well as having the results checked. The free comments were in line with this finding with one of the more common comments suggesting that the best combination was a human making the final decision but supported by AI.

There were several comments suggesting that AI should not be used at all. However, this was not the finding from the ranking analysis. It shows that whether it was human, or AI was only of minor importance compared to the error rate, and whether there was a “checker” or second reader. When considering the response to having a second reader, or checker, it was notable that participants valued having decisions being checked but noted that there was almost no difference as to whether the checker was human or AI.

There was a trend favouring rapid reporting.

There was no association between the views of the participants and their age, gender, level of education of whether they were undergoing treatment. There was a weak negative association between the use of AI as a checker and awareness about AI. This suggests that increasing awareness about AI may not necessarily improve acceptance.

There was a negative association between risk and education level, with higher education having a higher risk tolerance. There was a negative correlation between education level and error rate. Higher educational level is associated with both improved health outcomes, and a higher expectation of patient centric standard of care [15, 16].

Interestingly, a similar association has been noted in economics where an association between risk tolerance and education was also found [17]. Acceptable error rates in clinical application of AI needs careful consideration. In recent studies about AI in clinical settings, it has been shown that patients are less trusting of AI than they would be of a human rater, with higher standards in relation to error when considering AI [18]. This is interesting to consider and certainly needs to be incorporated into the design of clinical pathways, as well as communication around AI assisted decision making. The error rates chosen for this study represent reasonable thresholds discussed in the literature for both clinical domains and clinical applications for AI. For example, in radiology (6.8% acceptable error rate for AI, 11.3% for humans), Large Language Model based AI scribes (1–3%) [19], and human error in radiology diagnosis with postmortem confirmation of cases (20%) [20]. There is great variability across fields and techniques, and a balance of reasonable error thresholds were chosen to reflect this variance.

The study has a number of limitations. Firstly, the participants were self-selecting, and many found the ranking task difficult and was only available online, therefore excluding those without access to the internet. Secondly, only 48% of those who visited the site, completed the ranking task. This may have been due to the length or complexity of the survey especially for many of those participating who had visual loss. For those who had difficulty there were contact details for help, however, no participants used this. Despite the relatively low response rate, the advantage of online surveys however is the relatively large number of participants. Larger, more in depth studies will be needed to aid in the design of clinical pathways and oversight of AI systems in ophthalmology, the present sample represents a small percentage of the clinical population and as such has a limited generalisability.

Finally, the participants may not be representative of the general macular disease population, as 39% of the participants had a college or university level education and 72% were aware of AI and what it is.

## Conclusion

Patients with macular disease find AI to be acceptable in the assessment of retinal images. Patients prioritise accuracy, speed, and the presence of a checking mechanism when considering the adoption of AI in macular disease treatment pathways. These results should inform the development of patient focused policies for assured AI adoption.

## Summary

### What was known before

- AI is considered to present key opportunities in retinal medicine
- Detection and diagnosis of conditions can be made more efficient with the use of AI tools
- Radiology and oncology have explored patient views of AI decision making, but macular treatment decisions had not been explored

### What this study adds

- This study explores what patients view as acceptable for AI use in macular clinics
- This study adds the patient perspective of AI being acceptable in macular treatment decisions
- This study demonstrates a priority for accuracy from the patient perspective, with a tolerance for error in line with other clinical fields

## Supplementary information

Supplementary Information

## Data Availability

The data that support the findings of this study are not openly available due to reasons of patient sensitivity and are available from the corresponding author upon reasonable request. Data are located in controlled access data storage at the Macular Society.
