# Supplementary material for: The acceptability to patients with macular disease to have retreatment decisions being made by artificial intelligence
Source: Eye Open. 2026 Jan 19;2(1):4. doi: 10.1038/s44440-025-00011-7 (PMC12863649; doi:10.1038/s44440-025-00011-7)
Supplement: Supplementary file 1 — Supplementary Information [file 44440_2025_11_MOESM1_ESM.docx]

Supplementary Information

Artificial Intelligence (AI) is a new area of research that has significant implications on many aspects of life, and in particular health care. AI is a term used to describe using and training machines to mimic the human ability to think, learn and make decisions. In healthcare this could be used to train AI to assess health data, such as test results, enabling doctors to make quicker decisions and freeing up their time to treat patients. Research has already shown that AI has potential for use in the treatment of macular disease, by being able to interpret scans of the macula. With AI being used to detect and aid in diagnosis of conditions from these eye scans. This survey looks to understand what people with macular disease think of the use of AI in their treatment, and what factors are important to consider. This research will help researchers and practitioners develop better treatment approaches.

1. What is your age?
   1. 18-24
   2. 25-34
   3. 35-44
   4. 45-54
   5. 55-64
   6. 65-74
   7. 75-84
   8. 85-94
   9. 95+
2. What is your gender?
   1. Female
   2. Male
   3. Other
   4. Prefer not to say
3. If yes, are you currently receiving treatment (injections, laser, etc.
   1. Yes, anti-VEGF injections
   2. Yes, laser treatment
   3. Yes, other
   4. No
4. What is the highest level of education you have completed?
   1. Primary school
   2. Secondary school up to 16 years
   3. Higher or secondary or further education (A-levels, BTEC, etc.)
   4. College or university
   5. Post-graduate degree
   6. Prefer not to say

5. How aware are you of AI?

1. Very aware, understand and use AI
2. Aware of AI and what it is
3. Have heard of AI but do not understand what it is
4. Never heard of AI

Currently eye scans are assessed by humans who use the scans to diagnose conditions, and for those with wet AMD or Diabetic Macular Oedema, these scans are assessed to see how frequently you may require treatment. Imagine these scenarios, where there are different options on how your eye scans are assessed. We are looking to understand what factors are important to patients in different scenarios. These scenarios will look at four factors: - Human versus AI reader: Readers are the ones looking at the images from your scans and using what they see to make decisions. Such as whether the patient needs any treatment. This can be done by humans or by AI. - Error rate: With any decision there is a risk that the wrong choice is made which could lead to under or over treatment. A 1 in 10 error rate would mean 1 in every 10 decisions would be incorrect. - Time to receive results: This is the time it takes the reader to analyse the scan and make a decision. - Checking the results: Whether a second reader (human or AI) checks the images again. The aim is to see what factor has the strongest effect on your preferences and to make solving that our top priority. By ranking the 13 scenarios below in order (from 1 = most acceptable to 13 = least acceptable), we can work out the relative importance of each of these factors in your decision making. In performing this, we suggest starting by putting your favourites in first (say top 2 to 3) and then the same for the bottom two of three. You will be left with a bunch "in the middle" that seem all about equal. In that case, don't worry about them as they are already in a random order. That is absolutely fine as your answers will be averaged and we are looking for any consistent trends that stand out against the background.

7. Please rank these scenarios in order of preference, with 1 being most preferable, and 13 being least preferable

1. AI reader, with a 1 in 10 error rate, results in less than a day, and no one checking the results
2. Human reader, with a 1 in 20 error rate, results in 2 days, and no one checking the results
3. AI reader, with a 1 in 5 error rate, results in 2 days, and no one checking the results
4. Human reader, with a 1 in 10 error rate, results in 4 days, and no one checking the results
5. Human reader, with a 1 in 5 error rate, results in 4 days, and no one checking the results
6. Human reader, with a 1 in 5 error rate, results in less than 1 day, with another human checking the results
7. AI reader, with a 1 in 10 error rate, results in 2 days, with a human checking the results
8. Human reader, with a 1 in 5 error rate, results in 2 days, with another human checking the results
9. AI reader, with a 1 in 20 error rate, results in 4 days, with a human checking the results
10. Human reader, with a 1 in 10 error rate, results in 4 days, with another human checking the results
11. Human reader, with a 1 in 20 error rate, results in less than 1 day, with AI checking the results
12. Human reader, with a 1 in 10 error rate, results in 2 days, with AI checking the results
13. AI reader, with a 1 in 5 error rate, results in 4 days, with AI checking the results

8. Do you have any comments or views on AI in healthcare that you would like to share?
